# How Behavior of Nontarget Species Affects Perceived Accuracy of Scat Detection Dog Surveys

**DOI:** 10.1038/s41598-018-32244-1

**Published:** 2018-09-14

**Authors:** Karen E. DeMatteo, Linsey W. Blake, Julie K. Young, Barbara Davenport

**Affiliations:** 1 Washington University in St. Louis, Department of Biology & Environmental Studies, St. Louis, Missouri USA; 2 WildCare Institute at the Saint Louis Zoo, St. Louis, Missouri USA; 3 Nebraska Game and Parks Commission, Wildlife Division–Research, Analysis & Inventory Section, Lincoln, Nebraska USA; 4 U.S. Department of Agriculture, National Wildlife Research Center, Predator Research Facility and Department of Wildland Resources, Utah State University, Logan, Utah USA; 5 PackLeader Dog Training LLC, Gig Harbor, Washington, USA

## Abstract

Detection dogs, specially trained domestic dogs (*Canis familiaris*), have become a valuable, noninvasive, conservation tool because they remove the dependence of attracting species to a particular location. Further, detection dogs locate samples independent of appearance, composition, or visibility allowing researchers to collect large sets of unbiased samples that can be used in complex ecological queries. One question not fully addressed is why samples from nontarget species are inadvertently collected during detection dog surveys. While a common explanation has been incomplete handler or dog training, our study aimed to explore alternative explanations. Our trials demonstrate that a scat’s genetic profile can be altered by interactions of nontarget species with target scat via urine-marking, coprophagy, and moving scats with their mouths, all pathways to contamination by nontarget species’ DNA. Because detection dogs are trained to locate odor independent of masking, the collection of samples with a mixed olfactory profile (target and nontarget) is possible. These scats will likely have characteristics of target species’ scats and are therefore only discovered faulty once genetic results indicate a nontarget species. While the collection of nontarget scats will not impact research conclusions so long as samples are DNA tested, we suggest ways to minimize their collection and associated costs.

## Introduction

Developing management strategies for species, such as wide-ranging carnivores, requires population estimates and an understanding of habitat use. Several noninvasive techniques are available to gather these types of data but each have their particular advantages and disadvantages. Camera traps, for example, are effective for surveying carnivores^1–3^ but it is not always possible to identify individuals or even sex in species that are monomorphic and lack distinct scars or marks^4,5^. In addition, the technique’s dependence on attracting animals to a specific location (e.g., animal trail, open area, bait stations) may result in the failure to detect a species, individual, or sex class if they actively avoid these habitat types, avoid movements that overlap with potential competitors or predators, or are elusive in nature^6–8^. Further, camera traps may be difficult or impossible to use in areas of high human traffic due to risk of equipment theft or destruction resulting in data gaps. Similarly, hair snares can be used to survey carnivores but rely on collection structures to promote use and collect samples with sufficient DNA^9–12^. Success can vary depending on rate of hair shed or avoidance of collection devices due to presence of human odor^10^. Additionally, there is the potential that hairs from multiple individuals are collected as a single sample, permitting contamination between individuals^13^. The combination of two alternative noninvasive techniques, genetic analyses of scat located via detection dogs, overcomes these limitations.

Genetic analyses of scat switches the focus to locating evidence that animals inevitably leave behind with their natural behavior and movement patterns. For decades, scat has been used to define basic ecological parameters (e.g., diet, distribution, habitat use) for many species; however, these studies are dependent on an accurate identification of the donor species and misidentifications due to similarities in scat morphology, location of deposition, and odor could result in incorrect conclusions^14,15^. Advances in genetic techniques can remove this source of error. DNA extracted from scat can confirm donor species and be used to identify the sex and individual associated with each sample, allowing for more complex ecological and evolutionary questions to be addressed (e.g., population size, kinship relationships)^16–24^.

Detection dogs eliminate dependence upon a target species’ visitation rate to a specific area and a human’s reliance upon vision to locate scats. Detection dogs are domestic dogs (*Canis familiaris*) trained on a reward system to locate a particular odor or set of odors. Upon reaching the source of a target odor or the odor that has been paired with the reward, the detection dog is trained to give a specific response or action, which is consistent, reliable, and repeatable. While the trained response can differ among dogs depending on the training philosophy, it is not a random action or opportunistic response allowing it to be fundamental to the technique’s accuracy across odors. Over the last two decades, conservationists have used the extraordinary sense of smell and task-oriented focus of domestic dogs to locate various types of samples in a variety of habitats and from numerous species^25–34^. The olfactory search image of these dogs provides many advantages over the visual search image used by humans^35–38^. The dog’s ability to discriminate odor is not affected by visual appearance of samples. Dogs can also pinpoint a scat’s exact location whether it is exposed or masked by the environment, locate multiple target species within a search area while ignoring nontarget species, and cover a larger geographic area faster and more completely than humans working alone. In addition, the dog’s olfactory search image is not limited by the body size of the target species or the target species use or avoidance of specific habitat types. Further, unlike camera traps, the dog is able to work effectively in areas of higher human presence. These unique factors of detection dogs allow researchers to efficiently collect large sets of unbiased samples from targeted species that can be genetically analyzed to confirm species identity and distinguish individuals.

Even though detection dogs have a comparatively higher cost compared to camera traps or hair snares, this cost is offset by the dogs’ efficiency in detecting even rare target species^29,30,33,38^. However, one question that has not been fully addressed is why samples from nontarget species are collected in some detection dog surveys. Of studies that report the collection of scats from nontarget species, rates range from 3.7–44.6%^15,26,29,32,33,39–41^; however, few studies detail the species-identity of these nontarget samples. Nor do they confirm whether these nontarget samples were located by the dog as opposed to visually located by field personnel (B Davenport, unpublished data). Long *et al*.^42^ suggested incomplete handler or dog training may be the cause of nontarget sample collection. While this is a growing concern as the use of detection dogs in conservation studies expands, we explore additional explanations for the inadvertent collection of nontarget scats: (1) contamination of the genetic profile through urine deposition by a nontarget species on a target scat, (2) coprophagy of a target scat by a nontarget species, and (3) contamination of the genetic profile of a scat through saliva deposited on it when it is moved or mouthed by a nontarget species.

These alternative explanations most likely occur in territorial species that use urine and defecation marking behavior. Canids [e.g., coyotes (*Canis latrans*)] and felids [e.g., leopard (*Panthera pardus*)] are known for conspecific urine-marking for various ecological and social reasons^43–46^. We reason that similar markings occur between overlapping carnivores [e.g., cougar (*Puma concolor*) and coyote] with multiple individuals potentially marking a single location^47–51^. In addition, coprophagy has been reported in lagomorphs^52^, rodents^53^, and domestic dogs^54–56^. Many dog owners report their pets eagerly consuming the feces of domestic cats (*Felis catus*)^57^ and trained tracking dogs have been reported to opportunistically ingest cougar scat (TR Allen, personal communication). In wild canids, coprophagy has been primarily referenced in regards to territorial behavior in conspecifics (e.g., coyotes eating the feces of intruding coyotes)^58^ but may occur under other circumstances. Wild canids, such as coyotes, may similarly consume scats of other species, such as cougar, for reasons of territoriality or for the nutritional benefits associated with partially digested prey remains. Whether target scat has been urinated on, experienced coprophagy, or been mouthed and moved by a nontarget species, we propose the DNA of the nontarget species would overwrite or contaminate the DNA of the target species while leaving the olfactory signal of the target species intact.

**Figure 1:**
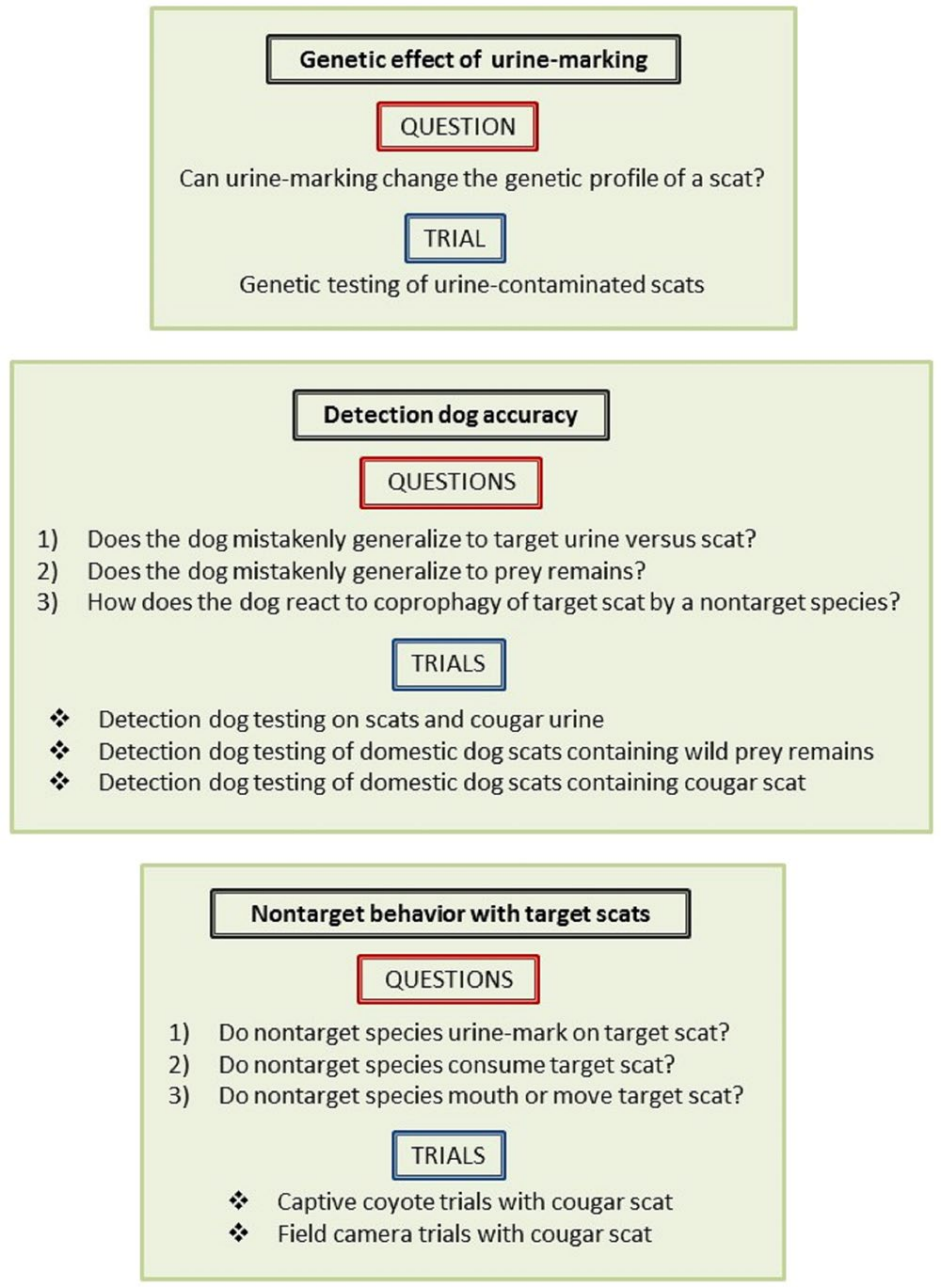
We conducted a multilevel investigation to determine if three proposed alternative explanations account for detection dogs (*Canis familiaris*) locating nontarget scats. First, we examined whether urine deposited on scat affects its genetic profile. Second, we tested the accuracy and olfactory search image of the detection dog in a set of outdoor trials. Third, we used trials with captive coyotes and wild carnivores to determine whether nontarget species urinate on, consume and defecate, or otherwise interact with target scats. Outlined are the three components with corresponding questions and relevant trials.

To determine if these alternative explanations account for detection dogs locating nontarget scats, we conducted a multilevel investigation (Fig. 1). First, we examined whether urine deposited on scat affects its genetic profile. Second, we tested the accuracy and olfactory search image of the detection dog in a set of trials. Third, we used trials with captive coyotes and wild carnivores to determine whether nontarget species urinate on, consume and defecate, or otherwise interact with target scats.

## Methods

### Alternative explanation 1: Genetic effect of uring marking

#### Genetic testing of urine-contaminated scats

Can urine-marking change the genetic profile of a scat? Fresh scats from six domestic cats were collected and dried for 72-hours in the sun, in order to mimic cougar scat samples collected in the field. Domestic cat as opposed to wild cougar samples were used to ensure the initial genetic profile matched the donor species by removing any potential effect of nontarget urine-marking that could occur with wild samples. The mixed urine of two domestic dogs was then applied to the scat in a way that mimicked the quantity of a typical urine-mark, which involves less urine than urination^57^ and determine to be ~2–3 ml (KE DeMatteo, unpublished data). The scat was then allowed to dry in the sun for another 24-hours.

The mtDNA was extracted from a swab of the each scat’s exterior using QIAGEN (Venlo, Netherlands) DNeasy TM DNA extraction kit following the protocol described in Vynne *et al*.^59^. Extractions were carried out in a room separate from the lab in which PCR amplifications were performed using dedicated equipment to prevent contamination. Negative controls (no scat material added to the extraction) accompanied each set of extractions and were used to in species identification PCRs to test for contamination. To identify species, a 110-base-pair (bp; 171-bp with primer) carnivore-specific region of mitochondrial cytochrome *b* gene^60^ with a modified version of the protocols and reagents^32,33,61,62^. Amplifications were performed on a MyCycler Thermal Cycler System (BioRad, Hercules, CA) in 25-µL volumes containing 2 µL of DNA extract^32,33^. To minimize the potential for contamination in all reaction, PCR reactions were performed in an Ultraviolet PCR Chamber and included negative controls (no DNA added). We edited and aligned sequences using Lasergene Seqman 8.1 (DNASTART, Madison, WI) and compare them with entries in GenBank using the Basic Local Alignment Search Tool (BLAST) to identify sequences that were identical with sample sequences.

### Alternative explanation 2: Detection dog accuracy

#### Detection dog

The detection dog used in all trials was a male Chesapeake Bay retriever with multiple seasons of work in Misiones, Argentina finding scat from five carnivores [jaguar (*Panthera onca*), cougar, ocelot (*Leopardus pardalis*), oncilla (*Leopardus tigrinus*), and bush dogs (*Speothos venaticus*)] and Nebraska, USA finding scat from a single carnivore (cougar). In both studies, the dog had been trained to locate the target species using scat from multiple individuals of both sexes on varying diets, both captive and wild. Upon reaching the source of a target odor or odor that he was trained to believe would result in his reward (i.e., play time with a tennis ball), the dog was trained to stop at the sample but not touch it, to remain quiet, and to hold the position until the handler arrived. With a strong positive response, the dog has a focused sniff of the odor followed by a quick physical shift to a raised ear set, strong tail wag, and direct stare at the handler. When the odor is located at a distance, this strong positive response is preceded by a dramatic shift in the dog’s animation, concentration, and attention to detail as it works the odor to its source. With a weak positive response, the dog has a slow but deliberate sniff followed by a gradual physical shift to looking at the handler with a moderate tail wag and little or no shift in ear set. With a negative reaction or no trained response, the dog acknowledges the presence of the odor through a toss or turn of the head in the direction of the sample but continues moving through the area with no demonstration of the specific actions trained to give with a target odor.

#### Detection dog testing of scats and cougar urine

Does the detection dog mistakenly generalize to target (cougar) urine versus scat? Urine was collected from the enclosures of captive male and female cougars in four zoos in the USA and frozen until use. The undiluted urine was thawed and mixed to give an odor profile that was not limited to one individual or a single sex for these trials. Target scats were collected from wild cougars in Nebraska, genetically confirmed at the USFS Rocky Mountain Research Station – National Genomics Center for Wildlife and Fish Conservation in Missoula, Montana, and frozen until use. Nontarget scats were collected from domestic and wild species. Because the goal of the test was to record the detection dog through observation, the source of nontarget scats was not a factor. Wild samples were collected in Nebraska, genetically confirmed as either bobcat (*Lynx rufus*) or a canid-species at USFS Rocky Mountain Research Station in Fort Collins, Colorado, and frozen until use. Domestic samples were freshly collected from multiple domestic cats of both sexes. They were used to increase the number of confirmed nontarget samples. All wild samples were tested with the detection dog prior to the trials to eliminate samples with a mixed odor profile due to coprophagy, urine-marking, or saliva.

Testing was conducted in three phases to determine to which odor(s) the detection dog was responding to. Each phase consisted of 2–3 trials with 10–14 odor stations in each. The reward system mimicked that used by the handler in the field; if the dog demonstrated a trained response and a scat (cougar or nontarget) was present, the dog was rewarded. If the dog had a trained response and no scat was present (i.e., urine only), the dog was not rewarded. The first phase tested the detection dog’s reaction to nontarget scat with and without cougar urine. Three odors were used: cougar scat, nontarget scat (bobcat, canid, or domestic cat), and nontarget scat with cougar urine. The cougar scat was used as a known target sample in order to provide a reward opportunity for the dog, a technique to maintain work drive in training and field surveys. The position of the cougar urine (2–3 ml) was alternated between directly on versus ~3 m away from the nontarget scat. The latter allowed a separation of the two target odors in the dog’s olfactory search image and an opportunity for the handler to see to which odor(s) the dog responded to. The order of the odors was varied with each trial with the stations consisting of 2–3 cougar scats, 4–5 nontarget scats, and 4–6 nontarget scats with cougar urine. Phase two aimed to understand the detection dog’s reaction to two odors from the target species: cougar urine versus cougar scat. The order of the odors was varied with each trial with the stations consisting of 3–4 cougar scat, 3–5 cougar scat with cougar urine, and 4–5 cougar urine only. The third phase examined the dog’s reaction to all of the four odors tested in the first two trials: cougar scat, nontarget scat, nontarget scat with cougar urine on or adjacent, and cougar urine only. The order of the odors was varied with each trial with the stations consisting of 2 cougar scats, 2–3 nontarget scats, 3–4 nontarget scats with cougar urine, 1–2 cougar scat with cougar urine, and 2–3 cougar urine only. The goal in this last phase was to test the dog’s current level of performance and any influence of target urine.

The three trials were conducted in an open area in St. Louis, Missouri where the dog’s trained response to samples could be clearly seen by the dog handler and observer. Different areas were used with each trial with wind and olfactory obstacles taken into account when establishing the trials. Whether the dog had a trained response (i.e., strong positive or weak positive) or had a negative reaction to each sample was recorded.

#### Detection dog testing of domestic dog scat containing prey remains

Does the dog mistakenly generalize to prey remains? Pieces of deer meat with hide and hair attached were obtained from fresh road kills in NW Nebraska. Hair was included in the samples in order to have a visual marker for prey presence in domestic dog scat and mimic the cougar scats, which will routinely have deer hair in their scat. Similar to those trial with scats (target and nontarget) and cougar urine, the goal in this phase was to test the accuracy of the dog’s olfactory search image and whether the dog was mistakenly generalizing to nontarget scat containing prey commonly consumed by cougar.

Four domestic dogs were fasted for 24 hours, presented with the deer pieces, and received their normal diet of commercial dog food 24 hours later. This 24 hour fast prior to the deer pieces follows the normal once per day feeding routine. All four dogs immediately consumed the offered deer pieces. All dog scats containing deer hair were collected the following day and marked with the dog’s name and time. Samples were frozen for later testing with the detection dog.

Testing of samples was conducted in an open area in St. Louis, Missouri where the detection dog’s trained response to target odor could be clearly seen by the dog handler and observer. In addition to the cougar scat at the start and end, two were placed in the middle. In between these cougar scats 1–3 domestic dog samples were placed. Whether the dog had a trained response (i.e., strong positive or weak positive) or had a negative reaction to each sample was recorded.

#### Detection dog testing of domestic dog scats containing cougar scat

How does the dog react to coprophagy of target scat (cougar) by a nontarget species (domestic dog)? Four presumed cougar scats were collected in the Pine Ridge area of NW Nebraska. These scats were genetically confirmed at Washington University in St. Louis as cougar using mtDNA extracted from a swab of each scat’s exterior following techniques detailed above and in DeMatteo *et al*.^32,33^.

Five domestic dogs were fasted for 24 hours, presented with cougar scats, and received their normal diet of commercial dog food 24 hours later. This 24 hour fast prior to the cougar scat followed the normal once per day feeding routine. All five dogs immediately consumed the offered cougar scats. All dog scats were collected for 48 hours after the presentation of cougar scats, marked with the dog’s name, date, and time, and frozen for later testing with the detection dog. Since the animals are privately owned, the trials were performed with permission from the owner who agreed that no physical harm was expected, as these dogs are known to consume cougar scats opportunistically on their own and receive regular anti-parasite treatments for this reason.

Testing of samples was conducted in the Pine Ridge area in NW Nebraska, in an open area where the detection dog’s trained response to samples could be clearly seen by the dog handler and observer. Prior to testing the dog’s reaction to the domestic dog samples, all samples were visually examined to determine what diet they contained: primarily cougar scat or primarily commercial dog food. A cougar scat was placed at the start, the middle, and the end. In between these cougar scats, three domestic dog scats containing cougar scat and one domestic dog scat with kibble were randomly placed. Whether the dog had a trained response (i.e., strong positive or weak positive) or had a negative reaction to each sample was recorded.

After the detection dog trial, mtDNA was extracted from a swab of each domestic dog scat’s exterior and a central portion to examine if coprophagy altered the genetic profile of ingested cougar scat. Techniques followed those detailed above and by DeMatteo *et al*.^32,33^.

### Alternative explanation 3: Nontarget behavior with target scats

#### Captive coyote trials with cougar scat

Do nontarget species (coyote) urine-mark, consume, mouth or move target (cougar) scat? Fourteen presumed cougar scats were collected in Nebraska, South Dakota, and Wyoming. These scats were genetically confirmed at Washington University in St. Louis as cougar using mtDNA extracted from a swab of each scat’s exterior following techniques detailed above and in DeMatteo *et al*.^32,33^.

Five pairs of captive coyotes at the National Wildlife Research Center’s Predator Research Facility in Millville, Utah were randomly selected to receive genetically confirmed cougar scats marked with orange glitter. The coyotes were fasted for 24 hours prior to the trial and received their normal daily food ration, marked with blue glitter, four hours after being presented with the cougar scats. An additional pair of coyotes served as the study control and received their regular food marked with orange glitter followed four hours later by their regular food marked with blue glitter. Each pair of coyotes was housed in a separate pen. Motion-activated trail cameras were used to monitor interactions between coyotes and cougar scats. All coyote scats containing glitter (orange and/or blue) were collected for 48 hours after the initial feeding, marked with the pen and coyote identity, and frozen for later testing with the detection dog. The trials were performed in accordance with the relevant guidelines and regulation and all animal handling protocols were approved by the Institutional Animal Care and Use Committee of the National Wildlife Research Center (QA-2753).

The testing of samples was done in the Pine Ridge area in Nebraska in an open area where the detection dog’s trained response to target odor could be clearly seen by the dog handler and observers. Prior to testing the dog’s reaction to the captive coyote scats, all samples from the five non-control pairs were visually examined to determine what color(s) of glitter they contained: primarily orange (i.e., cougar), primarily blue (i.e., regular diet), or orange/blue mix. The dog handler was blind to which samples were placed by the observers. Whether the dog had a trained response (i.e., strong positive or weak positive) or had a negative reaction to each sample was recorded.

#### Field camera trials with cougar scat

Do nontarget species (coyote) urine-mark, consume, mouth or move target (cougar) scat? Eight presumed cougar scats were collected in Nebraska, South Dakota, and Wyoming. These scats were genetically confirmed at Washington University in St. Louis as cougar using mtDNA extracted from a swab of each scat’s exterior following techniques detailed above and in DeMatteo *et al*.^32,33^. A single, genetically confirmed scat was placed in the center of three trail cameras at eight sites in Elkhurst, Missouri. At least one camera was set to take video-only. Each site was monitored for 14 days. Recorded photographs and video were classified for each species as: no response, smelling, moving and smelling, smelling and urine-marking, or smelling, moving and urine-marking. Due to the monomorphic characteristics in the observed species, individuals were not distinguished.

## Results

### Genetic testing of urine-contaminated scats

All six scats from domestic cats marked with dog urine were genetically identified as domestic dog, indicating a shift to the urine’s genetic profile.

### Detection dog testing of scats and cougar urine

In each of the trials across the three phases, the detection dog had a negative reaction (i.e., approached the location but left with no positive trained response given) to the scat from all nontarget species with (n = 24) and without cougar urine (n = 20). This was independent of the placement of cougar urine on or several meters from the nontarget scat. A negative reaction was noted with cougar-urine only (n = 16). A positive trained response was given with cougar scats with (n = 12) and without cougar urine (n = 20).

### Detection dog testing of domestic dog scat containing prey remains

Seven scats were collected from the four domestic dogs. Each of these scats clearly contained prey remains, which was evident by visible deer hair and a smooth, dark, non-kibble texture associated with the deer meat. In addition to these seven samples, four known cougar scat were included as known target samples. The detection dog had no response or a negative reaction to all seven domestic dog scats.

### Detection dog testing of domestic dog scats containing cougar scat

Six of the domestic dog scats from the five domestic dogs clearly contained cougar scat. This was evident by darker color, pungent smell, and visible prey contents (e.g., deer hair, bone fragments) compared to those scats containing commercial dog food (e.g., lighter color, texture, odor of kibble). Of the multiple scats containing commercial dog food, two were selected for testing.

In addition to these eight samples, three genetically confirmed cougar scats were included as known target samples. The detection dog had a strong positive trained response to all six dog scats containing cougar scat and to the three cougar scats. In contrast, a negative reaction was observed with dog scats containing only commercial dog food, including the detection dog urine-marking on one sample.

The six dog scats containing cougar scat were genetically identified as domestic dog, with no evidence of mixed or contaminated signal in the sequence, from both external and internal samples.

### Captive coyote trials with cougar scat

Sixteen scats were collected from the five test pairs and four scats were collected from the control pair of coyotes during the 48-hour collection period. While the goal of the remote cameras was to monitor the behavioral response of the six coyote pairs, information was limited because many coyotes picked up the cougar scats and moved them out of the field of view. However, cameras did document coprophagy, with at least one coyote eating the scat immediately. Other camera data confirmed other potential sources of nontarget DNA contamination with individual coyotes rubbing, rolling, picking-up, and urine-marking the cougar scat.

All six pairs of coyotes, including the control pair, produced scats with both orange and blue glitter indicating consumption of cougar scats in addition to their regular diet. For the control pair, one scat was primarily orange while three had a mixture of blue and orange. For the five test pairs, all scats contained some orange glitter but the proportion varied between a few flecks to a majority. In total, only six scats were primarily orange (i.e., cougar), while eight were primarily blue (i.e., regular diet) and two had mixed glitter.

We note that while we are confident in defining a scat as primarily orange, we are cautious about defining a scat as exclusively blue. The reason is associated with the narrow window (i.e., four hours) between the presentation of cougar scat and regular diet combined with the potential consumption of cougar scat at any time (i.e., when the normal diet was present). The presence of some orange glitter in all scats, both test and control pairs, suggests overlap in the consumption/digestion of the first and second feeding.

The detection dog had no reaction or a negative reaction to the four scats from the control pair. The detection dog had had a strong positive trained response on the six scats that were primarily orange glitter (i.e., cougar scat) and the two scats that were a mix of orange and blue glitter (i.e., mix of cougar scat and regular diet). However, the dog’s response with the primarily blue scats was mixed with 75% (n = 6) a weak positive trained response and 25% (n = 2) a negative reaction or no trained response.

### Field camera trials with cougar scat

A total of 139 visits were recorded at the eight sites with 41.0% being by non-furbearers [i.e., white-tailed deer (*Odocoileus virginianus*)] and 59.0% associated with furbearers. While only 7.2% of furbearer observations were coyote, 12.9% were associated with other canids [7.2% fox (*Vulpes vulpes* or *Urocyon cinereoargenteus*)] or felids (5.7% bobcat). The remaining 38.9% furbearer observations were either raccoon (*Procyon lotor*; 36.7%) or opossum (*Didelphis virginiana*; 2.2%).

All non-furbearer observations involved no contact and were recorded as no response (68.4%) or smelling (31.6%). While the majority of furbearer observations also involved no contact (63.4% no response and 28.1% smelling), 8.5% involved contact. Moving and smelling (2.4%) were observed in a single raccoon and single opossum. Smelling and urine-marking (4.9%) were observed in a single fox and three coyotes. Smelling, moving, and urine-marking were observed in a single coyote.

## Discussion

Our study confirmed that there are three viable, alternative explanations beyond errors in handler and/or dog training that can explain the collection of nontarget scats with detection dogs in some ecosystems (Fig. 1). First, we demonstrated that DNA in urine can alter the genetic profile of scat. DNA in urine has been identified as a valuable noninvasive alternative to the use of blood in humans^63^ and non-humans^64^. Modified DNA extraction techniques now allow long-term stored urine and even samples dropped in the field (e.g., in snow, on bark) to be used^65,66^. In fact, extracted DNA can determine when a urine-mark represents more than one individual, as would be expected with urine overmarking behavior in canids^65^. This potential for mixed samples emphasizes the need to use lab analyses that check for errors, especially when using cross-species polymorphic microsatellites loci to differentiate samples to species-level^67,68^.

Second, we demonstrated that a well-trained detection dog will not generalize odors in the olfactory search image but will locate scat that has a mixed olfactory signal. That is, the detection dog had a negative reaction or gave no trained response to urine from the target species or to nontarget samples containing prey that the target species also consumes. Extending this logic to the field, it would mean that collected nontarget scats were not coyote scat urine-marked by cougar or coyote scat containing prey the cougar also consumes. However, the collected nontarget scats could be cougar scat urine-marked by coyote or cougar scat that was ingested and defecated by a nontarget species. To the handler, these scats likely have some characteristics of the target species’ scat, so no question is raised until the genetic profile is returned. At this point, it might be impossible to view the original sample in its entirety, so the result is often confusion and an assumption that the dog was inaccurate as opposed the possibility that some target scats were urine-marked or experienced coprophagy. This could be what occurred in a detection dog study with the San Joaquin kit fox (*Vulpes macrotis mutica*)^26^. In areas where this endangered fox overlaps with the non-native red fox (*Vulpes vulpes*), 33% of the collected scats were nontarget. While this was attributed to the inability of the detection dog to ignore the overlapping, nontarget species, our study suggests it could be associated with urine marking or coprophagy altering the genetic profile of the target scats.

Finally, the trials with captive coyotes and camera traps provide additional support of these alternative explanations for the collection of nontarget scats in some detection dog surveys. Coprophagy was demonstrated both directly, with a camera recording a captive coyote consuming a cougar scat, and indirectly through varying levels of orange glitter found in coyote scats from all pens. As predicted, the detection dog had a positive trained response to coyote scats that were primarily composed of ingested cougar scat. Similarly, the detection dog had a negative reaction to the coyote scats from the control pair. We believe that the inconsistencies in the dog’s reaction to samples that appeared to be primarily composed of the regular coyote diet resulted from having too short of an interval (i.e., four hours) between the presentation of cougar scats and feeding of regular diet. This combined with the potential delay in the consumption of the cougar scats and potential overlap in cougar scat-regular diet consumption confounded the results. That is, there was not a clear line between cougar scat and regular diet in the time period when consumption/digestion could occur. In addition to coprophagy, the cameras captured captive and wild coyotes and other wild canids urine-marking and moving cougar scats in their mouths. As with urine, saliva is a viable, noninvasive alternative for DNA^69,70^; with even residual saliva proving valuable in free-ranging wildlife studies^71–73^.

In addition to errors with handlers and/or detection dog training, the proportion of nontarget scats collected in a survey is likely dependent on whether there are coexisting species that may interact with target scats. In areas where carnivores are being targeted but there is a lack of a generalist mesocarnivores, like the coyote, the proportion of nontarget scats may be low. This appears to be the case in Misiones, Argentina where carnivore studies with detection dogs have a low proportion of nontarget scats (3.7%) and the local mesocarnivore (i.e., crab-eating fox/*Cerdocyon thous*) consumes primarily fruit and small rodents^32,33^. In contrast, coyotes in Nebraska are widespread and cougar studies with detection dogs have a high proportion of nontarget scats (≥50%; S Wilson/Nebraska Game & Parks, unpublished data). The proportion of collected nontarget scats might also shift as a result of feral or free-roaming domestic dogs that may interact with target scats. While this study has focused on carnivores because of their specific behavioral repertoire, it is likely that what is seen here is happening among an unknown number of species. For example, other species may move scat with their hands (primates), snouts (pigs), or paws (bears). In all cases, it might be difficult or impossible to eliminate the collection of nontarget scats. As noted with coprophagy, the complete and rapid ingestion of target scat by a nontarget species can generate a scat whose content and structure appears similar to the target species, so the collector may misidentify it is a target scat until the genetic analyses determine otherwise. In addition, our trials demonstrate that swabbing the interior of the scat does not overcome the DNA contamination that occurs with coprophagy. However, with urine-marking it is possible to minimize the number of target scats contaminated with nontarget urine by collecting only the freshest scats (e.g., mucus layer or outer shell intact). In addition, scat studies in areas with nontarget overlap can benefit from an initial collection that “cleans” the area of old scat followed by collections every few days^74^ (KE DeMatteo, unpublished data) eliminating the old scat that may be urine-marked multiple times^47–51^. This sweeping of an area would be especially relevant in searches along roads or near human-modified structures, as these are associated with an increased frequency of urine-marking^75^.

Detection dogs likely find samples with a mixed olfactory profile (target and nontarget) but nontarget genetic profile because they are trained to locate an odor independent of the masking of that odor by physical position (e.g., underground, in vegetation), physical appearance, and other odors. The target odor equals their reward, so a trained dog will find that odor independent of whether it makes up 10% or 100% of the olfactory profile. This behavior follows the military and police dog training that laid a foundation for conservation detection dog work. In narcotic work, the goal is for a dog to find all drugs independent of whether they are obvious (analogous to a non-contaminated target scat) or if they are disguised by a stronger smelling substance and/or hidden somewhere out of sight (similar to a target scat urine-marked by a nontarget species). In other words, a well-trained detection dog will stay on target and not be deterred or confused by the presence of nontarget odors. While work has started to train detection dogs based on categories, such as the presence/absence of an accelerant with explosives^76^, with conservation work, this type of categorical training might be more difficult because there are multiple ways nontarget animals could contaminate target scats (e.g., urine, saliva, coprophagy). Furthermore, there is the added factor of general DNA quality, which is related to the age and exposure of the scat^77^.

Determining whether the collection of nontarget scats is due to our alternative explanations or dog/handler training errors is something that will need to be addressed on a case-by-case basis. While establishing a set of international training standards can minimize dog/handler training errors, the shifted genetic profiles from nontarget contamination (e.g., urine, coprophagy, saliva) are generally unavoidable. However, ensuring the use of field protocols that maximize the quality of DNA sampled can minimize this effect. While field personnel depend on detection dogs to locate scat samples, the decision on whether to collect and how to store samples is made by humans and directly affects subsequent analyses with the scat. DNA amplification success is affected by many factors, including the age of the sample, its exposure to the elements, and diet^78–81^. Success is also affected by where and when the DNA is collected and how it is stored. While the optimal method may vary with species, habitat, and type of sample^82^, swabbing the mucous layer on the scat’s exterior has proven effective in multiple carnivores^32,33,81,83^. However, given that where the sample is swabbed also has an affect^84^, collection of mucous in the field can maximize results by allowing personnel to know directionality of the scat (up versus down versus sides) and minimize loss of mucus when placed into a storage container (e.g., paper or plastic bag). Furthermore, collection from the bottom of the scat can potentially avoid areas that have been urine-marked and collect the DNA from the original donor.

The results of this study also have an effect beyond the questions surrounding detection dog surveys. That is, the implication of coprophagy within or between taxonomic groups (i.e., felids and canids) extends into other ecological questions, including potential avenues for parasite and disease transmission. As the boundary between wild and domestic populations becomes blurred, coprophagy may influence the spread of viruses (e.g., canine parvovirus)^85,86^ that are intermittently shed into feces or parasites that have animals as definitive hosts (e.g., echinococcus)^87,88^. However, coprophagy can cause confusion on true positive versus false positive infections, with the latter capturing parasite eggs that were ingested versus naturally shed^53,89^. While infections resulting from coprophagy likely have little effect on the consumer, these individuals function as transport hosts for parasite eggs and increase the exposure zone for many zoonotic infections (e.g., toxocariasis, echinococcosis)^88–91^. The findings of this study related to coprophagy presents new information that must be considered in studies on zoonotic diseases and general health of wild populations.

While this series of trials demonstrated three alternative explanations for the collection of nontarget samples in some detection dog studies, it would be remiss to ignore those nontarget samples that likely result from improper or inadequate training of the handler or dog^42^. Comparing the efficacy of detection dogs is difficult given the variability in study design and methodologies^92^. While positive reinforcement is relatively consistent across published studies, the approach taken to physically train the dog is varied or unreported. Establishing set protocols or guidelines for training detection dogs is not as straight forward as it might seem. Visual criteria (e.g., physical build, energy drive) used to select a detection dog can be narrowed but these characteristics must be balanced with other factors including, the handler’s personality, the target species, and the conditions in the study area^93,94^. When training the handler, it is important to include trials where sample location is known and unknown. The former allows the handler to identify those involuntary physical changes (e.g., ear set, tail wag, overall animation, change in concentrated sniffing) that occur when a detection dog encounters an odor that it has been trained to believe will result in a reward^95^, while the latter is important to ensure the handler is not unknowingly affecting change in the detection dog’s specific, trained response through nonverbal cues or physical prompting^96^. When selecting training samples it is important to maximize variety (e.g., diet, individuals, sex, age) for both targeted and nontargeted species^26,30–32,97,98^, as the chemical profile of scat can shift with each of these factors^99^. Training to ignore nontarget species is especially important when the target species is rare and overlaps with nontarget species in the same taxonomic group^33^. In both cases, it is important to reinforce and test the dog on scat from wild animals before commencing a survey to prepare the dog on the variety of diet shifts that a species can have (e.g, salmon-based, berry-based, and scavenger-based in bears). This is especially true of inexperienced dogs that have completed training but lack field experience, as they tend to be very specific and do not generalize across odors.

Enumerating wildlife populations is central to developing management actions, but in practice it can be difficult when dealing with uncommon, wide-ranging, or elusive species. Detection dogs have proven to be an effective tool in locating scats whose DNA can be genotyped and used in a mark-recapture framework to derive population estimates of target species^40,100^. While coprophagy, urine-marking, and mouthing and moving of scats may result in the collection of nontarget scats, researchers can minimize the impact to their conclusions so long as all samples are subjected to DNA testing. In order to reduce additional costs that nontarget scat collection incurs, it is important to consider ways to minimize their collection, including: maximize training sample variety, sufficient handler trials, “cleaning” of areas with nontarget overlap, practice of sample collection/storage that optimize DNA quality, and lab analyses that check for errors.
